# Supplementary material for: Response of essential oil hemp (Cannabis sativa L.) growth, biomass, and cannabinoid profiles to varying fertigation rates
Source: PLoS One. 2021 Jul 29;16(7):e0252985. doi: 10.1371/journal.pone.0252985 (PMC8320997; doi:10.1371/journal.pone.0252985)
Supplement: S1 Fig — Chromatograph examples of [a] a standard curve, [b] sample N-091 below the federal THC limit of 0.3%, [c] sample N-132 near the federal THC limit of 0.3%, and [d] sample N-173 above the federal THC limit of 0.3. (DOCX) [file pone.0252985.s001.docx]

S1 Fig. Chromatograph examples of [a] a standard curve, [b] sample N-091 below the federal THC limit of 0.3%, [c] sample N-132 near the federal THC limit of 0.3%, and [d] sample N-173 above the federal THC limit of 0.3%.

1. CS7 standard curve sample.

B) N-091sample (0.145% total THC).

C) N-132 sample (0.349 % total THC).

D) N-173 sample (0.483% total THC).

sample, [b]
