# Supplementary material for: Response of essential oil hemp (Cannabis sativa L.) growth, biomass, and cannabinoid profiles to varying fertigation rates
Source: PLoS One. 2021 Jul 29;16(7):e0252985. doi: 10.1371/journal.pone.0252985 (PMC8320997; doi:10.1371/journal.pone.0252985)
Supplement: S1 Table — Repeatability estimates calculated on an entry means basis. (DOCX) [file pone.0252985.s007.docx]

**S1 Table**. Percent variance explained by each model term of eq. 1. Repeatability estimates calculated on an entry means basis.

|  | **Variance Components** | | | | |  |
| --- | --- | --- | --- | --- | --- | --- |
| **Trait** | **Rep** | **Fertilizer Rate** | **Cultivar** | **Fertilizer-by-Cultivar** | **Error** | **Repeatability** |
| Kola Mass (g) | 2.06 | 45.62*** | 3.84** | 0.00 | 48.49 | 0.74 |
| Chaff Biomass (g) | 0.00 | 51.27*** | 2.44* | 0.87 | 45.41 | 0.63 |
| Total Biomass (g) | 0.00 | 45.79*** | 6.16*** | 0.64 | 47.42 | 0.81 |
| Flower Biomass (g) | 0.00 | 36.64*** | 8.63*** | 0.25 | 54.49 | 0.85 |
| Bucked Biomass (g) | 0.00 | 44.84*** | 5.93** | 0.22 | 49.01 | 0.81 |
| Stem Mass (g) | 0.00 | 44.04*** | 6.10** | 2.00 | 47.87 | 0.79 |
| Flower Harvest Index (%) | 2.45 | 8.05** | 6.79*** | 18.14** | 64.57 | 0.58 |
| Harvest Index (%) | 0.00 | 10.60*** | 0.00 | 17.60** | 71.80 | 0.00 |
| Percent Stem Weight (%) | 0.00 | 10.60*** | 0.00 | 17.60** | 71.80 | 0.00 |
| FWR:Chaff | 0.98 | 23.08*** | 3.45 | 0.00 | 72.49 | 0.63 |
| Max Stem Diameter (mm) | 0.00 | 64.11*** | 1.22* | 4.30* | 30.38 | 0.44 |
| SPAD 12/19/19 | 1.55 | 80.99*** | 2.06*** | 0.37 | 15.03 | 0.81 |
| Chlorophyll a (nmol mL^-1^) | 5.70 | 74.00*** | 0.21 | 0.00 | 20.00 | 0.28 |
| Chlorophyll b (nmol mL^-1^) | 3.60 | 81.50*** | 0.10 | 0.00 | 14.80 | 0.20 |
| Total Chlorophyll (a+b)(nmol mL^-1^) | 5.30 | 75.60*** | 0.20 | 0.00 | 18.80 | 0.28 |
| Plant height 31 DAS (m) | 0.00 | 0.95 | 28.05*** | 5.22 | 65.78 | 0.91 |
| Plant height 36 DAS (m) | 0.00 | 20.53*** | 23.48*** | 0.53 | 55.46 | 0.94 |
| Plant height 52 DAS (m) | 2.51 | 59.52*** | 8.72*** | 4.15 | 25.11 | 0.86 |
| Plant height 67DAS (m) | 1.07 | 73.14*** | 5.74*** | 0.44 | 19.62 | 0.90 |
| Plant height 77 DAS (m) | 0.00 | 75.09*** | 5.16*** | 0.04 | 19.70 | 0.90 |
| Plant height 99 DAS (m) | 0.00 | 73.91*** | 6.44*** | 0.30 | 19.35 | 0.92 |
| Plant height 114 DAS (m) | 0.17 | 74.33*** | 6.08*** | 0.00 | 19.42 | 0.92 |
| Cannabichromene (%) | 0.00 | 1.38 | 3.03 | 0.00 | 95.59 | 0.53 |
| Cannabidiol (%) | 2.79 | 3.10 | 5.91* | 0.00 | 88.20 | 0.71 |

**S1 Table.** Continued.

|  | **Variance Components** | | | | |  |
| --- | --- | --- | --- | --- | --- | --- |
| **Trait** | **Rep** | **Fertilizer Rate** | **Cultivar** | **Fertilizer-by-Cultivar** | **Error** | **Repeatability** |
| Cannabigerol (%) | 0.00 | 17.08*** | 1.75 | 5.09 | 76.09 | 0.37 |
| Cannabigerolic Acid (%) | 4.10 | 9.65** | 24.62*** | 6.79 | 54.80 | 0.90 |
| 9-Tetrahydrocannabinol (%) | 0.00 | 4.51* | 10.22*** | 8.64 | 76.63 | 0.74 |
| Tetrahydrocannabinolic Acid (%) | 3.21 | 21.38*** | 9.10*** | 0.00 | 66.31 | 0.83 |
| Weibull Asymptote (m) | 0.00 | 73.99*** | 6.48*** | 0.02 | 19.52 | 0.92 |
| Weibull Growth Rate | 0.00 | 41.26*** | 0.41 | 6.13 | 52.21 | 0.14 |
| Weibull Inflection Point (d) | 0.00 | 62.95*** | 0.57* | 6.77** | 29.71 | 0.23 |
| Kola Density (g cm | 0.00 | 43.77 | 8.05 | 0.00 | 48.18 | 0.86 |
| Max.AGR (m d^-1^) | 0.76 | 70.67*** | 2.78** | 1.09 | 24.71 | 0.76 |
| Max.AGR.DAS (d) | 0.00 | 84.96*** | 0.39 | 0.48 | 14.17 | 0.45 |
| Half.Max.AGR (m d^-1^) | 0.76 | 70.67*** | 2.78** | 1.09 | 24.71 | 0.76 |
| D1.Half.Max (d) | 0.00 | 76.79*** | 0.62 | 1.08 | 21.51 | 0.44 |
| D2.Half.Max (d) | 0.00 | 46.81*** | 0.15 | 10.52** | 42.52 | 0.05 |
| Half.Max.Duration (d) | 0.00 | 4.41** | 0.65 | 17.43** | 77.51 | 0.11 |
| CBD:THC ratio | 0.00 | 0.90 | 7.19 | 0.00 | 91.91 | 0.74 |
| Total Potential THC (% dry mass) | 0.00 | 17.66*** | 13.97*** | 0.00 | 68.36 | 0.88 |
| Total Potential CBD (% dry mass) | 1.50 | 30.20*** | 10.96*** | 0.00 | 57.34 | 0.87 |
| Total Potential CBG (% dry mass) | 0.33 | 14.45*** | 21.88*** | 7.21 | 56.13 | 0.89 |
| THC Harvest (g plant^-1^) | 0.00 | 35.20*** | 10.85*** | 2.93 | 51.02 | 0.85 |
| CBD Harvest (g plant^-1^) | 0.00 | 37.28*** | 8.76*** | 2.25 | 51.71 | 0.83 |
| CBG Harvest (g plant^-1^) | 0.00 | 25.91*** | 12.48*** | 5.73 | 55.88 | 0.83 |

*** Significance at α<0.05**

**** Significance at α<0.01**

*****Significance at α<0.00**
