# Supplementary material for: Response of essential oil hemp (Cannabis sativa L.) growth, biomass, and cannabinoid profiles to varying fertigation rates
Source: PLoS One. 2021 Jul 29;16(7):e0252985. doi: 10.1371/journal.pone.0252985 (PMC8320997; doi:10.1371/journal.pone.0252985)
Supplement: S2 Table — Relative trait reductions indicate the reduction of the trait at or above the ECW threshold. Percentages in parentheses indicated the percent reduction of the trait relative to the 50 ppm N fertilizer treatment (EC = 0.54). (DOCX) [file pone.0252985.s008.docx]

**S2 Table.** Irrigation water salinity thresholds (EC_W_) which caused statistically significant (Tukey’s LSD α=0.05) reductions in the trait of interest compared to the 50 ppm N fertilizer treatment (EC=0.54). Relative trait reductions indicate the reduction of the trait at or above the EC_W_ threshold. Percentages in parentheses indicated the percent reduction of the trait relative to the 50 ppm N fertilizer treatment (EC=0.54).

| **Trait** | **EC_W_**  **threshold** | **EC_W_**  **fertilizer**  **treatment** | **Relative trait**  **reduction** |
| --- | --- | --- | --- |
| Plant height (31 DAS) | ns | ns | ns |
| Plant height (36 DAS) | ns | ns | ns |
| Plant height (52 DAS) | 2.85 | 600 ppm N | 6 cm (12%) |
| Plant height (67 DAS) | 2.85 | 600 ppm N | 21 cm (21%) |
| Plant height (77 DAS) | 2.22 | 450 ppm N | 16-31 cm (13-25%) |
| Plant height (99 DAS) | 1.59 | 300 ppm N | 14-35 cm (11-26%) |
| Plant height (114 DAS) | 1.59 | 300 ppm N | 14-35 cm (11-26%) |
| Weibull asymptote | 1.59 | 300 ppm N | 15-36 cm (11-26%) |
| Weibull growth rate | 2.85 | 600 ppm N | 0.4 (13%) |
| Weibull inflection point | 2.85 | 600 ppm N | 3.4 d (5%) |
| Max AGR | 2.22 | 450 ppm N | 0.4 -0.8 cm d^-1^ (14-30%) |
| Max AGR DAS | 2.22 | 450 ppm N | 3-5 d (5-9%) |
| Half max AGR | 2.22 | 450 ppm N | 0.2-0.4 cm d^-1^ (14-30%) |
| D1 | 2.85 | 600 ppm N | 5.7 d (19%) |
| D2 | ns | ns | ns |
| D2-D1 | ns | ns | ns |
| Total biomass | 2.85 | 600 ppm N | 84 g (59%) |
| Floral biomass | 2.22 | 450 ppm N | 20-35 g (34-60%) |
| Chaff biomass | 2.85 | 600 ppm N | 26 g (55%) |
| Total bucked biomass | 2.85 | 600 ppm N | 62 g (58%) |
| Stem mass | 2.22 | 450 ppm N | 11-22 g (30-60%) |
| Bucked biomass HI | ns | ns | ns |
| Flower HI | ns | ns | ns |
| Percent Stem Weight | ns | ns | ns |
| FWR:Chaff | ns | ns | ns |
| Max stem diameter | 2.22 | 450 ppm N | 0.2-0.4 cm (15-28 %) |
| SPAD 12/19/19 | 0.54 | 50 ppm N | 7.4 (14%) |
| Chlorophyll a | ns | ns | ns |
| Chlorophyll b | ns | ns | ns |
| Total chlorophyll (a+b) | ns | ns | ns |
| Cannabichromene (CBC) | ns | ns | ns |
| Cannabidiol (CBD) | ns | ns | ns |
| Cannabidiolic acid (CBDA) | 1.59 | 300 ppm N | 2.8-4.5 % (27-44%) |
| Cannabigerol (CBG) | ns | ns | ns |
| Cannabigerolic acid (CBGA) | ns | ns | ns |
| 9-Tetrahydrocannabinol (THC) | ns | ns | ns |
| Tetrahydrocannabinolic acid (THCA) | 1.59 | 300 ppm N | 0.11-0.18 % (24-40 %) |
| Total potential THC | 2.22 | 450 ppm N | 0.15-0.18 % (34-42 %) |
| Total potential CBD | 1.59 | 300 ppm N | 2.4-4.1 % (25-43 %) |
| Total potential CBG | 2.22 | 450 ppm N | 0.05 % (29%) |
| CBD:THC | ns | ns | ns |
| CBD Yield | 0.96 | 150 ppm N | 2.14-4.40 g/plant (37-76%) |
| CBG Yield | 2.22 | 450 ppm N | 0.08-0.09 g/plant (64-74%) |
| THC Yield | 1.59 | 300 ppm N | 0.10-0.19 g/plant (37-75%) |

ns: No significant reductions in trait across fertilizer treatments.
EC_W_: Irrigation water salinity thresholds
