# Supplementary material for: Response of essential oil hemp (Cannabis sativa L.) growth, biomass, and cannabinoid profiles to varying fertigation rates
Source: PLoS One. 2021 Jul 29;16(7):e0252985. doi: 10.1371/journal.pone.0252985 (PMC8320997; doi:10.1371/journal.pone.0252985)
Supplement: Attachment — Submitted filename: ReviewerResponses.docx [file pone.0252985.s010.docx]

Check carefully the whole MS, missing of spacing (many), absence of spacing in different symbols such as “>” “<” “=” “≥” including figure captions and figures.

*That you for the comment. We have reviewed the manuscript and made the appropriate corrections.*

Line 63: Include “the” before the word “best”

*Corrected.*

Line 183: ---at 4°C, 3220 ×g---. Use space after “4” and after “×”

*Corrected.*

Line 194: ---fertilizer/ ECW---. Delete the space after “/”. Change small “x” to the cross symbol “×”. Follow this style for whole MS e. g., line 275, 307, 309,

*Corrected.*

Figures 4-6: Check the error bars. It seems too high. Also, seems that the lettering doesn’t in accordance with error bars.

*Thank you for the comment we have double check the figures and the error bars are correct, indicating the 95% confidence intervals. The connecting letters were also checked, show no sign of differing from the error bars, and are consistent with the original JMP output.*
